# Categorization of *Escherichia coli* outer membrane proteins by dependence on accessory proteins of the β-barrel assembly machinery complex

**DOI:** 10.1016/j.jbc.2023.104821

**Published:** 2023-05-15

**Authors:** Nakajohn Thewasano, Edward M. Germany, Yuki Maruno, Yukari Nakajima, Takuya Shiota

**Affiliations:** 1 Organization for Promotion of Tenure Track, University of Miyazaki, Miyazaki, Japan; 2 Frontier Science Research Center, University of Miyazaki, Miyazaki, Japan

**Keywords:** protein folding, protein assembly, gram-negative bacteria, outer membrane, beta-barrel protein, beta-barrel assembly machinery (BAM) complex, membrane protein, *Escherichia coli* (*E. coli*)

## Abstract

The outer membrane (OM) of gram-negative bacteria is populated by various outer membrane proteins (OMPs) that fold into a unique β-barrel transmembrane domain. Most OMPs are assembled into the OM by the β-barrel assembly machinery (BAM) complex. In *Escherichia coli*, the BAM complex is composed of two essential proteins (BamA and BamD) and three nonessential accessory proteins (BamB, BamC, and BamE). The currently proposed molecular mechanisms of the BAM complex involve only essential subunits, with the function of the accessory proteins remaining largely unknown. Here, we compared the accessory protein requirements for the assembly of seven different OMPs, 8- to 22-stranded, by our *in vitro* reconstitution assay using an *E. coli* mid-density membrane. BamE was responsible for the full efficiency of the assembly of all tested OMPs, as it enhanced the stability of essential subunit binding. BamB increased the assembly efficiency of more than 16-stranded OMPs, whereas BamC was not required for the assembly of any tested OMPs. Our categorization of the requirements of BAM complex accessory proteins in the assembly of substrate OMPs enables us to identify potential targets for the development of new antibiotics.

Gram-negative bacteria contain an outer membrane (OM), a permeable barrier, and an inner (cytoplasmic) membrane. Outer membrane proteins (OMPs), a major component of the OM, are responsible for maintaining integrity and performing various functions in the OM (1, 2). A common characteristic of OMPs is the transmembrane domain, a β-barrel that folds into a single large cylinder of antiparallel β-sheets (3). In Gram-negative bacteria, the β-barrel domains of all OMPs are composed of an even number of β-strands, providing a thermodynamically stable antiparallel network for the β-barrel. OMPs can be broadly classified into two groups (i) monopolypeptide barrels (*e.g.*, OmpC) (4) or (ii) multipolypeptide barrels (*e.g.*, GspD) (5). Currently, monopolypeptide OMPs vary from 8- to 36-stranded, with species able to homo-oligomerize into monomers, dimers, or trimers (6, 7, 8, 9, 10, 11, 12, 13).

OMPs are synthesized in cytosolic ribosomes and then cross the inner membrane *via* the Sec translocon (14). The periplasmic chaperones SurA, Skp, or DegP help transport to the OM or rapidly degrade in the case of mislocalization (15). Most monopolypeptide OMPs are inserted into the OM by the multisubunit molecular machine, known as the β-barrel assembly machinery (BAM) (16). The central component of the BAM complex is BamA, a member of the Omp85 protein superfamily, which is an essential protein conserved across bacterial lineages and eukaryotic organelles (17, 18). Another essential subunit, BamD, is conserved across all gram-negative bacteria (19). Furthermore, BamB, BamC, BamE, and BamF have been identified as subunits of the BAM complex (19). They are necessary for efficient BAM complex function as “accessory subunits” but are not essential for bacterial viability. The composition of accessory subunits of the BAM complex varies in different subclasses of Gram-negative bacteria. For instance, the β-proteobacteria *Neisseria meningitidis* does not have BamB homologs (20).

In *Escherichia coli* (*E. coli*), the BAM complex is a heteropentameric complex composed of BamA, BamB, BamC, BamD, and BamE. The *E. coli* BamA, an OMP, contains five repeat polypeptide transport-associated (POTRA) domains upstream of the β-barrel transmembrane domain (21). The POTRA domains are exposed in the periplasm and interact with four lipoproteins. Recent studies utilizing structural approaches have revealed the molecular mechanisms of the *E. coli* BAM complex from multiple atomic resolution structures (22, 23, 24, 25, 26). The periplasmic domain of the BAM complex forms a funnel-like structure mainly through the POTRA domains of BamA and BamD. All accessory subunits interact with the outer wall of the funnel. BamB interacts directly with the POTRA domain of BamA, whereas BamC and BamE bind to BamD, forming the BamCDE unit. The β-seam, which is between the first and last strand of the β-sheet of BamA (termed the lateral gate), takes an open and closed formation and acts as a catalytic site (25, 26, 27, 28). The formation of an antiparallel β-hairpin between the N-terminal strand of the lateral gate and C-terminal strand of the substrate proteins initiates the insertion step. Some structural studies have captured assembly intermediates, in which BamA and substrate proteins form a fully membrane-inserted hybrid barrel interlinked binding at the lateral gate. Prior to membrane insertion, the interior wall moiety of the BamD funnel interacts with the substrate protein and promotes partial β-sheet folding (29).

At present, all roles by the subunits BamA and BamD are deemed essential systems required for all OMPs, regardless of the character of the protein. In contrast, the roles of accessory proteins have not been assigned in this system. Several studies have revealed the function of these accessory proteins. BamB stabilizes the assembly precinct formed by the localization of multiple BAM complexes, enabling rapid oligomerization of homotrimeric OMPs such as OmpC by assembling multiple molecules in close proximity (30). BamE stimulates conformational changes in the BAM complex (31), and BamB and BamE double deletion *E. coli* strains became synthetically lethal (32), indicating that both of these functions are important for assembly. However, a comprehensive understanding of how accessory proteins are involved in the assembly of various OMPs is lacking.

In this study, we employed an *in vitro* reconstitution assay using an isolated mid-density membrane fraction (EMM; *E. coli* mid-density membrane). EMM is the membrane fraction of sonicated *E. coli* collected by high-speed centrifugation among differential centrifuges (low-speed, high-speed, and ultra-high-speed). We isolated EMMs from the accessory protein deletion and wildtype (WT) strains. To categorize the substrates according to the requirement of accessory proteins, we selected seven different monopolypeptide barrel-forming substrates, containing 8- to 22-stranded monomers, homodimers (two-pores), or homotrimers (three-pores). BamB was necessary for efficient assembly of OMPs with 16 or more β-strands and homotrimeric substrates. While the lack of BamC did not impair substrate assembly efficiently, BamE assisted in the proper assembly of all substrates, regardless of size or oligomeric state. Analysis of the state of the BAM complex showed that the lack of BamE destabilized the BAM complex formation, whereas BamB and BamC had limited effects. As all protein assemblies were impaired by BamE deletion, it is implied that BamE is important for the stable connection of BamD to BamA. Our analysis demonstrates the role of accessory proteins in the BAM complex, which can facilitate the assembly of a wide variety of substrates. These findings clarify which subunits should be targeted for the development of assembly inhibitors of pathogenic OMPs.

## Results

### Reconstitution of assembly using isolated mid-density membranes

**Figure 1:**
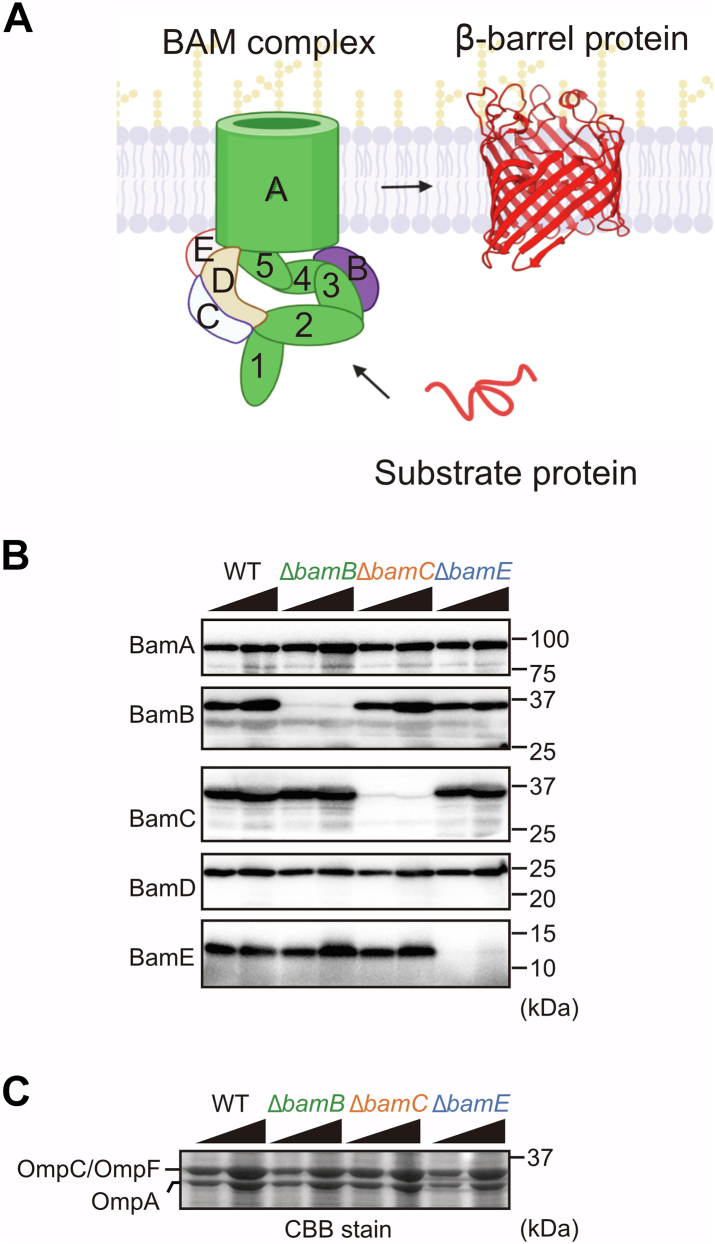
**β-barrel assembly machinery (BAM) complex steady-state levels in isolated *E. coli* mid-density membrane (EMM).***A*, schematic model of the BAM complex and outer membrane protein assembly. BamA (*green*) with the other accessory proteins, BamB (*purple*), BamC (*light blue*), BamD (*orange*), and BamE (*pink*). Numbers indicated the polypeptide transport-associated domains of BamA. The substrate protein was assembled by the BAM complex into the β-barrel structure. *B*, EMM fractions were isolated from strains indicated strains, wildtype (WT) indicated BL21(DE3)∗. Total proteins in EMM were analyzed by SDS-PAGE and immunoblotting with indicated accessory protein antibodies. Wedges correspond to either 2 μg or 6 μg of total EMM protein load. *C,* The steady state of major porins levels in the same EMM samples as in (*B*) were observed by CBB stain SDS-PAGE. Wedges correspond to either 24 μg or 72 μg of total EMM protein.

Gram-negative bacteria have envelope stress response systems that maintain OM integrity. Since the deletion of the accessory proteins of the BAM complex induces the σE stress response, which regulates gene expression of the OMPs (periplasmic or chaperones) (33), it is difficult for *in vivo* analysis to directly assess the functions of the accessory subunits. To overcome this problem, we employed an *in vitro* reconstitution assay using EMM containing intact BAM complexes. In this study, we deleted accessory proteins by gene disruption using a kanamycin cassette in BL21 (DE3)∗ as the parental strain. We isolated EMMs from each strain and then normalized them to the total protein level by the UV method. A comparison of the protein levels of subunits of the BAM complex at EMM equal total protein levels showed that deletion of accessory protein genes lost only itself but did not significantly affect the level of the other subunit proteins of the BAM complex (Fig. 1*B*). BamA levels in each EMM were not statistically different (Fig. S1), and we normalized the total protein level to assess the assembly efficiency. Adjusting the protein level of EMM between WT and mutant strains enabled us to compare the function of the BAM complex with minimal secondary effects, such as gene expression. EMM is composed of a native membrane formed by lipopolysaccharides and phospholipids, as well as an intact BAM complex. Thus, using EMMs isolated from each accessory protein deletion strain enabled us to assess the function of the BAM complex, which loses each accessory protein. Moreover, the levels of the major porins, OmpA, OmpC, and OmpF, in each EMM observed by the Coomassie brilliant blue (CBB) stain (34), corresponded to previous studies showing that Δ*bamB* or Δ*bamE* decreased the steady-state of major porins in the membrane fractions (Fig. 1*C*) (32).

For substrate proteins, we synthesized radiolabeled protein using methionine- or cysteine-containing radioactive isotopes, sulphur-35 (^35^S), by rabbit reticulocyte lysate. To assess only the assembly reaction performed by the BAM complex, all substrate proteins for the EMM assembly assay lacked the N-terminal SEC signal sequence (14). Previously, we established the reconstitution of assembly into EMM of five different OMPs: major porin (OmpA) (6), autotransporter (EspP) (8), major porins (OmpC and OmpF) (9), and maltoporin (LamB) (10, 35). In this study, we additionally established the assembly of two more OMPs: the integral OM protease (OmpT) (7) and iron uptake system (CirA) (11). This allows for a comparison of the assembly efficiency for a diverse range and characteristics of OMPs (Table 1).

**Table 1:** Characteristics range of outer membrane proteins (OMPs) used in this study

Protein name	Structure (side)	Structure (top)	Number of β-strand	Oligomeric state	PDB ID reference
OmpA			8	Monomer (Dimer)	1BXW (6)
OmpT			10	Monomer	1I78 (7)
EspP			12	Monomer	3SLJ (8)
OmpC			16	Trimer	2J4U
OmpF			16	Trimer	3O0E (9)
LamB			18	Trimer	1AF6 (10)
CirA			22	Monomer	2HDF (11)

The 3D structure of the OMPs used in this study in side view and top view were provided with the characteristics of each OMPs in two categories, number of β-strands, and oligomeric state. This was followed by PDB ID access code.

### BamE, but not BamB and BamC, is responsible for the full efficiency of 8- to 12-stranded OMP assembly

**Figure 2:**
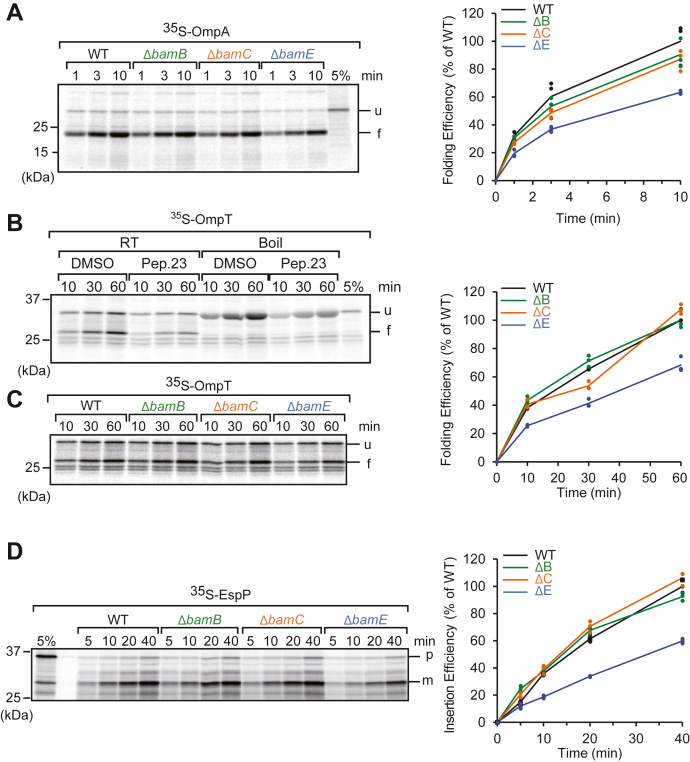
**Less than 12-stranded outer membrane proteins (OMPs) require BamE for efficient folding.***A*, an ^35^S-labeled OmpA, 8-stranded OMP, was synthesized by rabbit reticulocyte lysate, then incubated with *E. coli* mid-density membranes (EMMs) isolated from cells lacking an accessory BAM complex. At the indicated time, the EMM samples were shifted onto ice to halt folding reaction. Assembly was determined by heat modifiability and SDS-PAGE followed by radioimaging. 5% indicated 5% of input of ^35^S-labeled OmpA into each timepoint. The right graph indicates the amount of folded form of OmpA, and 10 min of the wildtype (WT) was set to 100%. *B*, OmpT (10-stranded OMP) folding was inhibited by β-signal mimic, peptide 23. ^35^S-labeled OmpT was incubated with EMMs in the presence or absence of peptide 23 and for the indicated times. The EMM samples were shifted onto ice to halt folding reaction. Samples were divided into half and subjected to SDS-PAGE after boiled or nonboiled (RT). *C*, ^35^S-labeled OmpT was incubated with mutant EMM for the indicated times, and the assembly assay was performed as in (*B*). Folding efficiency is indicated in the right graph as in (*A*). *D*, assembly of EspP (12-stranded OMP) with mutant EMMs. Proteinase K was added to removed uninserted protein and the passenger domain. Proteins were analyzed by SDS-PAGE and radioimaging. (p) and (m) indicate precursor and mature form, respectively. The right graph indicates insertion efficiency from the amount of mature form.

We analyzed the requirement of accessory proteins for OmpA, which has the fewest β-stranded OMPs (8-stranded OMPs) (Fig. 2*A*). As OmpA has a periplasmic exposed domain at the C terminus, the last strand of the barrel domain of OmpA is not located at the C terminus of proteins like typical OMPs. OmpA forms a dimer in the OM under specific conditions (36). OmpA was assigned as a monomeric OMP because OmpA dimerization requires a C-terminal domain. The insertion of the OmpA fold into the EMM *via* the BAM complex has been previously validated by a competition assay using the peptide of the range of the final strand of the OmpC, termed peptide 23, which inhibits the substrate recognition of the lateral gate of BamA (29, 35). We incubated EMMs with ^35^S-labeled OmpA at 30 °C and then shifted on ice to halt the assembly reaction. OmpA folding efficiency was monitored *via* “heat modifiability” (37, 38, 39, 40). Properly folded OMPs, including OmpA, can maintain a folded structure in the presence of sodium dodecyl sulfate (SDS) if it is not thermally denatured by boiling, but unfold when boiled. This was observed *via* SDS-polyacrylamide gel electrophoresis (PAGE) by the faster migration of folded OmpA compared to the unfolded form. Densitometry and comparison of folded OmpA showed that the assembly efficiency of OmpA in the EMM isolated from the BamE deletion strain was reduced to approximately 60% compared with other EMMs (Fig. 2*A*). OmpT is a 10-stranded monomeric OMP; while OmpT was used as a substrate protein for the reconstitution assay using purified BAM complex-embedded proteoliposomes (41), this study is the first to use OmpT for the EMM assembly assay. We validated whether the folding of OmpT into EMM occurred *via* the BAM complex following peptide 23 inhibition (Fig. 2*B*). Faster migration of OmpT significantly decreased in the presence of peptide 23, indicating that OmpT was assembled into the EMM *via* the BAM complex. We then compared the requirements of accessory proteins (Fig. 2*C*). The EMM isolated from the Δ*bamE* strain decreased the folding efficiency of OmpT to approximately 60%, as OmpA did.

EspP, an autotransporter, contains two domains: the N-terminal passenger domain and C-terminal 12-stranded β-barrel domain. The passenger domain traverses the OM *via* a transiently formed asymmetric barrel of BamA with EspP during the assembly of the barrel domain, and then, the passenger domain is cleaved off by self-cleavage (42). This reaction can be reproduced with a short passenger domain, with most of it removed (43, 44). In this study, similar to our previous reports, we used a passenger domain with only 76 amino acids remaining close to the barrel domain, termed the precursor form. Because the barrel domain of EspP, which is properly inserted into EMM and removed the passenger domain, the mature form, became proteinase K (PK) resistant, we analyzed insertion efficiency by the amount of the PK-resistant self-cleaved form of EspP. The insertion efficiency of EspP into Δ*bamE* also decreased to approximately 60% compared to that of the others (Fig. 2*D*). These results suggest that among the accessory proteins, only BamE is needed for the efficient insertion of 8- to 12-stranded monomeric OMPs into the OM.

### Assembly of homotrimeric OMPs was enhanced by BamB and BamE, but not BamC

**Figure 3:**
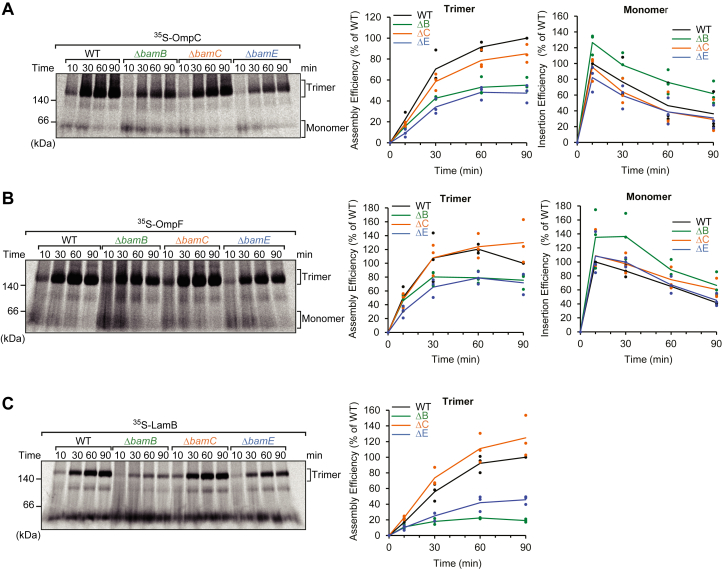
**Assembly assay of homotrimeric outer membrane proteins (OMPs).***A*, ^35^S-labeled OmpC was incubated with *E. coli* mid-density membranes (EMM) for 10, 30, 60, or 90 min. Protein complexes were solubilized with detergent and analyzed by BN-PAGE and radioimaging. The trimeric and monomeric forms were observed at 140 kDa and at the *bottom* of the gel, respectively. Graphs showed the assembly efficiency of trimeric (*Left*) and monomeric (*Right*) form in each accessory protein deletion strains compared to the wildtype (WT). The amount of trimer at 90 min in the WT set to 100%, and monomer at 10 min set to 100%. *B*, ^35^S-labeled OmpF assembly assay, described as in (*A*). *C*, ^35^S-labeled LamB were incubated with EMM for 10, 30 or 90 min and then analyzed by BN-PAGE and radioimaging. The trimeric form and monomeric form were present above140 kDa. The *line chart* showed the assembly efficiency of trimeric form of LamB in each accessory protein deletion strains compared to WT (BL21) as in (*A*).

OmpC and OmpF are 16-stranded β-barrel porins with a molecular weight of approximately 37 kDa (per monomer) and form stable homotrimers in the OM. As this homotrimer is resistant to SDS, OmpC, OmpF, or LamB can be observed at approximately 70 or 160 kDa by SDS-PAGE for heat modifiability, compared to the faster migration observed in monomeric OMPs (45, 46). Alternative methods to visualize trimeric OmpC or OmpF at a clearer resolution are through n-Dodecyl-β-D-maltoside (DDM) solubilization and analysis by blue native (BN)-PAGE. A previous study showed that membrane-inserted OmpC immediately forms trimers in WT EMM (30). In this study, we assessed the ability of each BAM complex based on the efficiency of OmpC and OmpF to assemble trimers, as observed by BN-PAGE (30, 35). Trimerization of OmpC was impaired by approximately 50% by Δ*bamB* and Δ*bamE* (Fig. 3*A*). The contribution of BamB to the trimerization of OmpC has been previously reported (30). A previous study revealed that BamB is responsible for forming the cluster of the BAM complex, termed assembly precinct, and suggested that the role of assembly precinct is rapid oligomerization by inserting multiple OmpC into the membrane in close proximity. As in the previous study, the lack of BamB impaired rapidly forming trimers, and as such, we observed an increase in monomer form at the bottom of the gel. The monomer in the *bamB* deletion EMM exhibited an extended half-life compared with other oligomeric states (Fig. 3*A*). The *bamE* deletion EMM decreased both trimer and bottom gel signal (monomer) compared to the WT, indicating that the lack of BamE impaired the assembly of OmpC. OmpF, a porin similar to OmpC, also had a requirement for accessory proteins similar to OmpC (Fig. 3*B*). The lack of BamB decreased the trimeric form and accumulated the monomer. The absence of BamE decreased the signals of both the monomer and trimer.

Maltoporin LamB is an 18-stranded trimeric OMP; therefore, we reconstituted the EMM assembly assay using this protein (35). Similar to OmpC and OmpF, LamB assembly was stimulated by both BamB and BamE (Fig. 3*C*). However, while BamB and BamE contributed equally to the assembly of OmpC and OmpF, the requirement of BamB for LamB assembly was significantly stronger than that of BamE. These results suggest that BamB plays an important role in the assembly of OMPs greater than 16-stranded, and this trend became stronger with an increase in the number of β-strands. Moreover, BamE contributed to the efficient insertion of trimer OMPs as well as monomeric small OMPs.

### Folding of 22-stranded monomeric OMPs was enhanced by both BamB and BamE

**Figure 4:**
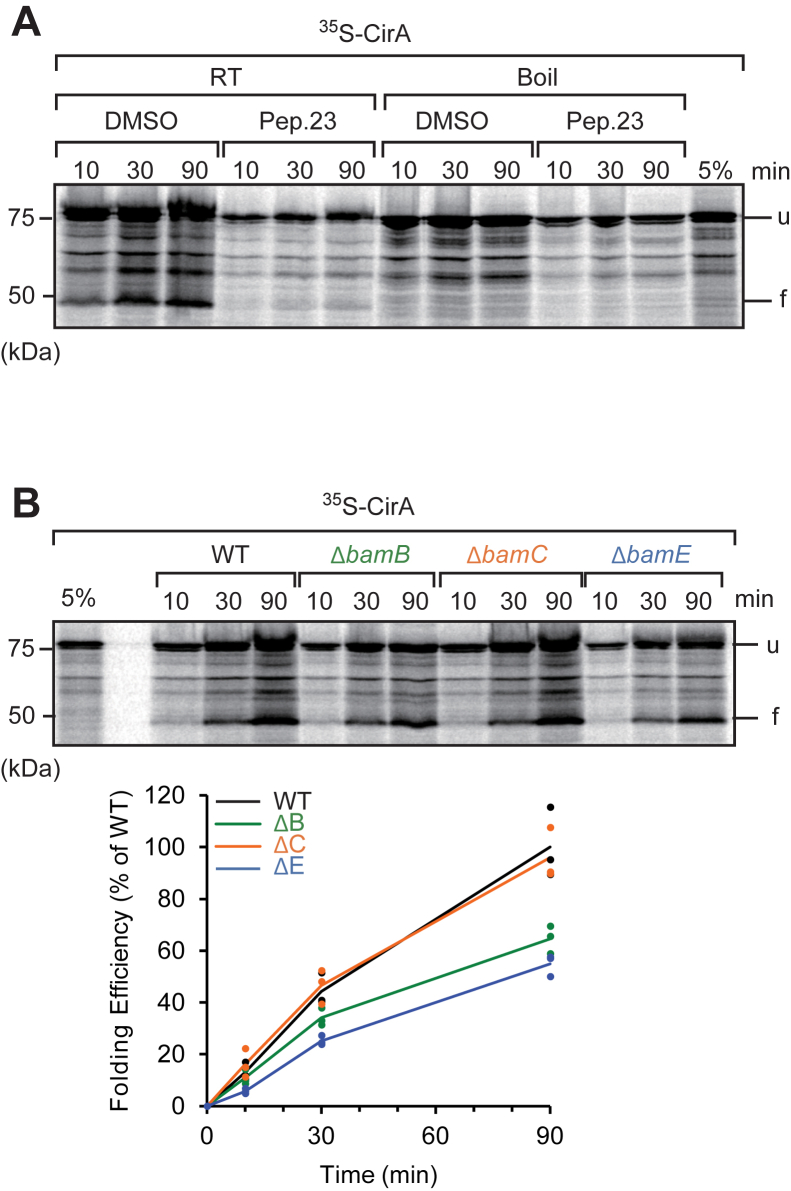
**Twenty two-stranded monomeric outer membrane proteins (OMPs) need both BamB and BamE for efficient folding.***A*, the folding of the CirA (22-stranded) OMP was confirmed by peptide 23 competition and heat modifiability, as in Figure 2*B*. (u) and (f) indicate to unfold and folded form. *B*, ^35^S-labeled CirA was synthesized by rabbit reticulocyte lysate and incubated with each accessory protein deletion strain *E. coli* mid-density membranes (EMM). At the indicated times, the EMM samples were shifted to ice to halt the folding reaction. Proteins were analyzed using SDS-PAGE and radioimaging.

CirA, an iron uptake system from iron bound to catecholate siderophores, is a 22-stranded monomeric OMP (47). We established the EMM assembly assay using CirA as a substrate and validated the folding of CirA in EMM *via* the BAM complex using the peptide 23 competition assay (Fig. 4*A*). The addition of peptide 23 strongly inhibited the formation of faster migration, the folded form of CirA. This folded form was not observed after boiling the samples, indicating that the EMM assembly assay can accurately analyze the folding of CirA in the membrane. Folding of CirA was significantly decreased in EMMs isolated from Δ*bamB* or Δ*bamE* cells compared to that in WT cells (60%) (Fig. 4*B*). The decrease in folding efficiency due to BamE loss was consistent across all OMPs. BamB stimulated the assembly of more than 16-stranded OMPs, regardless of their oligomeric state.

### BamE is responsible for the stability of the BamA–BamD interaction

**Figure 5:**
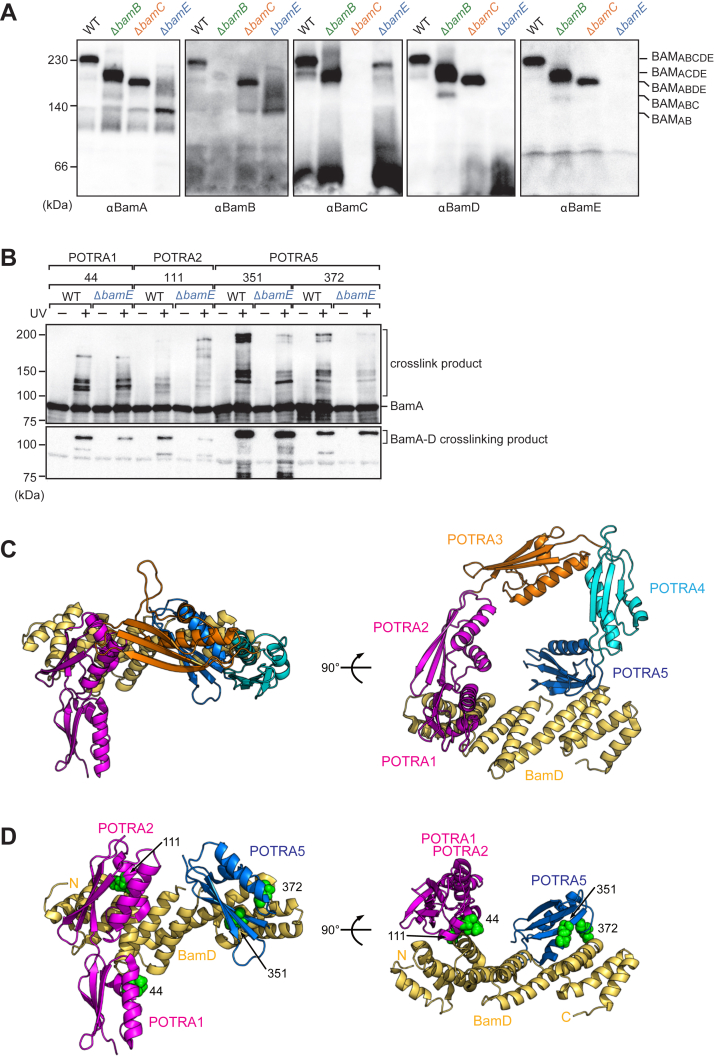
**Analysis of the β-barrel assembly machinery (BAM) complex stability caused by lack of accessory proteins.***A*, blue native (BN)-PAGE Western blot analysis of BAM complex, *E. coli* mid-density membrane (EMM) proteins were solubilized by DDM, then clarified by BN-PAGE and detected by immunoblotting against α-BamA, α-BamB, α-BamC, α-BamD, and α-BamE antibodies. *B*, the BPA were introduced into BamA at the amino acid positions, 44, POTRA 1; 111, POTRA 2; 351 and 372, POTRA 5, respectively. Cells were irradiated with or without UV (+ or −), and then BamA and crosslinked products were purified with Ni-NTA. Eluted fractions were analyzed by SDS-PAGE and immunoblotting with anti-BamA or anti-BamD antibodies. *C*, structure (5D0O) of the POTRA domains of BamA and BamD. *D*, BPA introduced positions (*green*) were plotted on tertial structure of the POTRA 1, 2 (*magenta*), and 5 (*blue*) with BamD (*yellow*). The *left* is a horizontal viewpoint with respect to the membrane; the *right* is a 90° rotation of the *left*. BPA, *p*-benzoyl-_L_-phenylalanine; POTRA, polypeptide transport-associated.

To understand the molecular mechanisms of the accessory proteins involved in assembly, we analyzed the effects of deleting each accessory protein on the architecture of the BAM complex. As we previously described, the steady-state levels of all subunits of the BAM complex were not impaired by the deletion of each subunit (Fig. 1*B*). We then analyzed the stability of the BAM complex against detergent solubilization by BN-PAGE and immunoblotting using antibodies against each subunit of the BAM complex (Fig. 5*A*). In the WT EMM, all subunits, BamA, BamB, BamC, BamD, and BamE, were observed at 250 kDa, indicating that all subunits formed the BAM_ABCDE_ complex. In Δ*bamB* and Δ*bamC*, all subunits except the deleted subunit were detected at the same location, indicating that the other subunits formed a complex (Δ*bamB*, BAM_ACDE_; Δ*bamC*, BAM_ABDE_). Interestingly, in Δ*bamE* EMM, BamA stably interacted with only BamB and partially interacted with BamC, but BamD did not interact with BamA. BamC and BamD mainly migrated to the bottom of the gel, implying that BamD and BamC dissociated from the central BamA subunit. This suggests that the lack of bamE influences the interaction of BamA with the BamCDE unit.

Inhibition of all OMP assembly by Δ*bamE* EMM is likely to be caused by a lack of the central substrate assembly system by the essential subunits BamA and BamD. However, if the BamA–BamD interaction was completely lost, the growth of the Δ*bamE* strain would be more severe. An alternative possibility is that BamE contributes to the stability of BamA and BamD interactions, and BamA-BamD binding without BamE was sensitive to DDM solubilization. To determine whether the dissociation of BamC and BamD from BamA in the EMM of Δ*bamE* was due to detergent solubilization or was occurring in the native state, we employed *in situ* site-specific photocrosslinking using the photoreactive unnatural amino acid, *p*-benzoyl-_L_-phenylalanine (BPA) (48). Based on reported structures, we selected appropriate positions to introduce BPA into BamA for crosslinking with BamD. According to the atomic resolution structures of the BAM complex, the N-terminal or C-terminal regions of BamD interact with POTRA 2, or POTRA 5 of BamA, respectively (Fig. 5*C*) (22, 23, 24, 49). In the WT strain, BamA was crosslinked to BamD at the amino acid positions 44, POTRA 1; 111, POTRA 2; and 351 and 372, POTRA 5 (Fig. 5*B*). We then performed the same crosslinking experiment with the Δ*bamE* strain. The cross-linked products between BamA (BPA) and BamD were formed at all four positions in both the strains (Fig. 5, *B* and *D*). However, the cross-linked products between BamD and POTRA1 (residue 44) or POTRA2 (residue 111) of BamA significantly decreased in the bamE deletion strain, but not POTRA5 of BamA. In other words, POTRA5 of BamA was able to bind to the C-terminal side of BamD without BamE, but not POTRA1 and 2 of BamA to the N-terminal side of BamD. These results suggest that the loss of one of the two binding regions between BamA and BamD due to the lack of BamE decreases the stability of the BamA and BamCD units. Therefore, BamE contributes to all OMP assemblies by stabilizing the interaction between the essential subunits, BamA and BamD.

## Discussion

**Figure 6:**
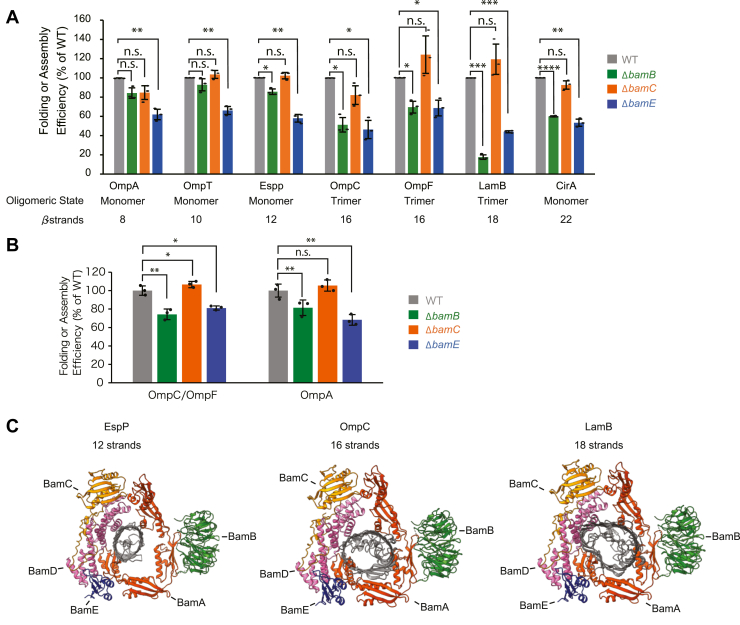
**Efficiency of folding, insertion, or assembly of various outer membrane proteins (OMPs) into wildtype (WT) or accessory proteins deletion mutant in both *in vitro* (EMM assembly assay) and *in vivo*, and schematic model of this study.***A*, folding, insertion, and assembly efficiencies are the amount of OMPs assembled after the longest incubation time of each EMM assembly assay normalized by the amount of BamA in the EMM used for each experiment. Assembled OMPs into WT EMM were set to 100%. The oligomeric state and number of the β-strands were described at the *bottom*. All experiments were performed three technical replicates (n = 3). Statistical significance was indicated by following signals: n.s. (not significant), *p* > 0.05; ∗*p* < 0.05; ∗∗*p* ≤ 0.01; ∗∗∗*p* ≤ 0.001. Exact *p* values, OmpA folding, WT *versus* Δ*bamB*: *p* = 0.05433; WT *versus* Δ*bamC*: *p* = 0.09414; WT *versus* Δ*bamE*: *p* = 0.00998; OmpT folding, WT *versus* Δ*bamB*: *p* = 0.24765; WT *versus* Δ*bamC*: *p* = 0.34359; WT *versus* Δ*bamE*: *p* = 0.00735; EspP insertion WT *versus* Δ*bamB*: *p* = 0.01938; WT *versus* Δ*bamC*: *p* = 0.42695; WT *versus* Δ*bamE*: *p* = 0.00452; OmpC assembly, WT *versus* Δ*bamB*: *p* = 0.01171; WT *versus* Δ*bamC*: *p* = 0.12075; WT *versus* Δ*bamE*: *p* = 0.01519; OmpF assembly, WT *versus* Δ*bamB*: *p* = 0.01949; WT *versus* Δ*bamC*: *p* = 0.22142; WT *versus* Δ*bamE*: *p* = 0.03229; LamB assembly, WT *versus* Δ*bamB*: *p* = 0.00040; WT *versus* Δ*bamC*: *p* = 0.22396; WT *versus* Δ*bamE*: *p* = 0.00018; CirA folding, WT *versus* Δ*bamB*: *p* = 0.00009; WT *versus* Δ*bamC*: *p* = 0.13647; WT *versus* Δ*bamE*: *p* = 0.00314. *B*, summary of the densitometry of major porin in the indicated EMMs. SDS-PAGE and Coomassie brilliant blue (CBB) staining performed three technical replicates (n = 3). Statistical significance was indicated by signals as in (*A*). Exact *p* values, an amount of OmpC/F, WT *versus* Δ*bamB*: *p* = 0.00118; WT *versus* Δ*bamC*: *p* = 0.02389; WT *versus* Δ*bamE*: *p* = 0.02056; an amount of OmpA, WT *versus* Δ*bamB*: *p* = 0.00995; WT *versus* Δ*bamC*: *p* = 0.05804; WT *versus* Δ*bamE*: *p* = 0.00294. *C*, schematic model of funnel formed by POTRA 1-2 and 5 of BamA and BamD with the substrate protein EspP (*left*), OmpC (*middle*), and LamB (*right*), the POTRA domain of BamA are shown in *orange*, BamB in *green*, BamC in *yellow*, BamD in *pink*, BamE in *blue*, and substrate protein in *gray*. PDB ID; BAM complex, 6V05, Espp 2QOM, OmpC 2JLN, and LamB 1MPM. BAM, β-barrel assembly machinery; EMM, *E. coli* mid-density membrane; POTRA, polypeptide transport-associated.

In this study, we analyzed the role of accessory proteins of the BAM complex in the assembly of OMPs using an *in vitro* reconstitution assay of seven substrate proteins, an EMM assembly assay, and *in vivo* analysis of steady-state protein levels and protein complex formation. The absence of BAM complex accessory protein did not influence the abundance of BamA in the EMMs when normalized by unit total protein. BamA showed no statistically significant difference between the WT and the mutants lacking BAM complex accessory proteins (Fig. S1). However, to eliminate the influence of BamA variation on folding, insertion, or assembly efficiency, we normalized each experimental result by the BamA amount. This provided a more accurate measure of the true assembly activity (Fig. 6*A*). Specifically, we found that in Δ*bamB* EMM, the substrates with statistically greater assembly inhibition (<70% of WT) were OMPs with 16 or more β-strands (OmpC, OmpF, LamB, and CirA). *In vivo* analysis showed that the reduction in the steady-state levels of OmpC/F (75% of WT) was greater than that of OmpA (80% of WT) in Δ*bamB* EMM (Fig. 6*B*). In contrast, all substrates showed a statistically significant effect in Δ*bamE* EMM. The *in vivo* analysis showed Δ*bamE* EMM had a decrease in the monomeric protein, OmpA, by 65% and trimeric proteins, OmpC/F, by 80% of WT EMM (Fig. 6*B*). The BamC was not required for OMP assembly in either *in vivo* or *in vitro* analyses. Our findings suggest that the lack of accessory proteins has a significant impact on assembly efficiency, independent of slight variations in the abundance of BamA.

BN-PAGE analysis and *in situ* site-specific photocrosslinking demonstrated that BamE contributed to the stabilization of the binding of BamA and BamCDE units (Fig. 5, *A* and *B*). According to structures of the BAM complex, BamE interacts concurrently with POTRA5 of BamA and the C-terminal region of BamD, located outside of the funnel-like structure of the periplasmic domain of the BAM complex (22, 23, 24, 49). The funnel is formed by the POTRA domains of BamA on one side and BamD on the other, with contact sites at the N-terminal and C-terminal regions of BamD with POTRA 2 or POTRA 5 of BamA, respectively (Fig. 5, *C* and *D*). The crosslinking approach demonstrated that BamE stimulated the interaction between POTRA2 and the N-terminal side of BamD. BN-PAGE analysis revealed that the interaction between BamA and BamCDE units could not endure the solubilization of detergent without BamE. Previous studies using biochemical approaches and neutron reflectometry analysis have revealed that BamE plays important roles in the configuration of the POTRA domain and extracellular loop (31, 50). The structure of the assembly intermediate of the EspP-BAM complex revealed that the substrate protein interacted simultaneously with both essential subunits (BamA and BamD) during the assembly reaction. Taken together, our findings imply that BamE may stimulate the stability of binding between the two essential subunits for efficient substrate protein transfer by controlling the configuration of the BAM complex.

The EMM assembly assay clearly demonstrated that BamB was involved in the assembly of more than 16-stranded OMPs but not in the insertion or folding of less than 12-stranded OMPs. Some assembly of intermediate structures of the BAM complex have revealed that the substrate partially folds antiparallel β-sheets during binding to BamD in the periplasm (26, 29, 35). Considering the partial folding of OMPs in the periplasm and substrate binding from the BamD side, we located each substrate in the interior funnel of the periplasmic domain of the BAM complex (Fig. 6*C*). Using EspP, the largest BamB-independent OMP, as a substrate, a gap was observed between the substrate and the surface of the interior wall of the BamB-interacting side (Fig. 6*C*). When 16- or 18-stranded OMPs, OmpC, and LamB were modeled, both the BamB and BamD sides of the interior walls were in close proximity. This structural insight may support our findings related to BamB, with a possible novel signal that binds to BamB or to this region of the POTRA domain. A previous report has shown that double deletion mutants of BamB and BamE or BamC in *E. coli* became synthetically lethal (32). If BamB is involved in a region other than the C-terminal side of the substrate recognized by the BamCDE unit, this is a possible explanation for the synthetically lethal double deletion mutants.

Given the immediate dangers associated with antimicrobial resistance (51), several recent drug screens have identified lead compounds that exert lethal effects on gram-negative bacteria (52, 53, 54, 55, 56, 57). However, most of these candidates enter the lateral gate of BamA, and a diversity of antibiotic targets is essential. Our findings suggest that BamB is a potential antibiotic target for regulating the assembly of more than 16-stranded OMPs. According to the distribution of accessory proteins in the subclass of Proteobacteria analyzed by the hidden Markov model, BamB and BamE exist in α-, β-, and γ-proteobacteria, but not δ- and ε-proteobacteria (19). Further understanding of the molecular mechanisms of BamE may enable the development of subclass-limited antibiotics. Moreover, the EMM assembly assay has the advantage of easy addition of candidate compounds, a characteristic of *in vitro* systems. The establishment of the model substrates performed in this study will allow for the development of inhibitor screening for a variety of OMP substrates.

## Experimental procedures

### Bacterial strains and plasmids

*E. coli* strains, plasmids, and primers used in this study are listed in Tables S2–S7. The accessory protein deletion strains utilized in this study were designed and generated by Lithgow Lab. In brief, the kanamycin-resistance gene cassette was amplified by PCR with the 5′ and 3′ flanking regions of the gene of interest in KEIO collections (Δ*bamB*, Δ*bamC*, or Δ*bamE*) (58). PCR products were transformed into *E. coli* BL21 (DE3)∗ cells harboring pKD46 (59) and then selected transformants by LB (1% tryptone, 1% NaCl, and 0.5% yeast extract) containing 30 μg/ml kanamycin. Gene disruption was validated by PCR and immunoblotting of target gene protein products. We used the SLiCE method to ligate DNA fragments for plasmid construction (60).

### Preparation of the *E. coli* EMM

EMM was prepared as previously described (30). Briefly, *E. coli* cells were cultured to an *A*_600_ of 0.8 to 1.0 in LB. Cells were harvested by centrifugation (4500 rpm, 7 min, 4 °C). Cell pellets were resuspended in sonication buffer (50 mM Tris-HCl, pH 7.5, 150 mM NaCl, and 5 mM EDTA) and disrupted by sonication on ice. The disrupted cell mixtures were centrifuged (3000 rpm, 5 min, 4 °C), and the supernatant was transferred to a new tube and clarified by centrifugation (11,000 rpm, 10 min, 4 °C). The pellet was resuspended in SEM buffer (250 mM sucrose, 10 mM Mops-KOH, pH 7.2, and 1 mM EDTA), snap-frozen in liquid nitrogen, and stored at −80 °C. Protein concentrations of EMM were calculated by *A*_*280*_ measurements of 100 times diluted in 0.6% SDS (*A*_280_ = 0.21, set to 10 mg/ml).

### Steady-state protein level and protein complex analysis

To check steady-state protein levels, EMM pellets were resuspended in SDS-PAGE sample buffer (125 mM Tris-HCl, pH 6.8, 2 mM EDTA, 2%(w/v) SDS, 1%(w/v) sucrose, 0.03% (w/v) bromophenol blue, 1% (v/v) β-mercaptoethanol). The samples were heated at 98 °C for 10 min, centrifuged at 12,000 rpm for 5 min at 25 °C, then subjected to SDS-PAGE. Afterward, the levels of the accessory proteins were analyzed by immunoblotting with anti-BamA, anti-BamB, anti-BamC, anti-BamD, and anti-BamE antisera. The major porin protein levels were analyzed by SDS-PAGE gel staining with CBB.

To check the BAM complex, EMM pellets were solubilized in 1.5% DDM containing BN-PAGE lysis buffer (25 mM imidazole-HCl, pH 7.0, 50 mM NaCl, 50 mM 6-aminohexanoic acid, 1 mM EDTA, 7.5% (w/v) glycerol) on ice for 20 min. Solubilized proteins were clarified by centrifugation at 15,000 rpm for 10 min at 4 °C, mixing with 20x BN-PAGE sample buffer (4.0% (w/v) CBB G-250, 100 mM 6-aminohexanoic acid), and subjected to BN-PAGE. After BN-PAGE electrophoresis, the gel was incubated in denaturing buffer (200 mM Tris-HCl, pH 7.2, 4% (w/v) SDS, 0.5% (v/v) β-mercaptoethanol), and then the proteins were transferred to a PVDF membrane. PVDF membrane transferred proteins were washed with 100% methanol and immunoblotted with anti-BamA, anti-BamB, anti-BamC, anti-BamD, and anti-BamE antisera.

### *In vitro* transcription and translation

Each substrate protein for the EMM assembly assay was synthesized using rabbit reticulocyte lysate (Promega). Linearized DNA fragments for *in vitro* transcription were amplified from the proteins of interest with the Sp6 RNA polymerase binding site cloned into the pTnT vector as a template by PCR using a pair of primers pTnT-f and pTnT-r. The mRNA was synthesized by Sp6-RNA polymerase using linearized DNA as a template (Takara). The transcribed mixture was added to the reticulocyte lysate mixture (Promega) in the presence of ^35^S-methionine and ^35^S-cysteine (PerkinElmer). We added an amino acid mixture (minus methionine) to one-tenth of the Promega protocol volume.

### OmpA EMM folding assay

For each time point, EMM 75 μg pellets were resuspended in 100 μl assembly assay buffer (pH 7.2, 10 mM Mops-KOH, 2.5 mM KH_2_PO_4_, 250 mM sucrose, 15 mM KCl, 5 mM MgCl_2_, 2 mM methionine, 5 mM DTT, 1% w/v BSA, 0.09% v/v Triton X-100) supplemented with 10 μl ^35^S-labeled OmpA. The assembly mixtures were incubated at 30 °C for 1, 3, or 10 min. At the indicated time points, the mixtures were shifted on ice to halt the folding reaction. EMMs were then harvested by centrifugation at 14,000 rpm for 5 min at 4 °C and resuspended in SEM buffer. Samples were collected by centrifugation at 14,000 rpm for 5 min at 4 °C, resuspended in SDS-PAGE sample buffer, and analyzed by SDS-PAGE and radioimaging.

### OmpT EMM folding assay

For one time point, EMM 30 μg pellets were resuspended in 75 μl assembly assay buffer supplemented with 7.5 μl ^35^S-labeled OmpT. The assembly mixtures were incubated at 30 °C for 10, 30, or 60 min. At the indicated time points, the mixtures were placed on ice for 5 min. The EMMs were then harvested by centrifugation and resuspended in SEM buffer twice using a sonication bath. OmpT-folded EMM proteins were collected by centrifugation, resuspended in SDS-PAGE sample buffer, and analyzed by SDS-PAGE and radioimaging. For the peptide 23 competition assay of OmpT, 200 μM peptide 23 (synthesized by Mimotopes) was added to the assembly assay buffer containing EMM prior to the addition of ^35^S-OmpT, and folding assays were performed as described above.

### EspP EMM insertion assay

The EspP insertion assay was performed as described previously. Briefly, for one time point, EMM 75 μg pellets were resuspended in 100 μl assembly assay buffer and supplemented with 10 μl ^35^S-labeled EspP. The assembly mixtures were incubated at 30 °C for 5, 10, 20, or 40 min. At the indicated time points, the mixtures were placed on ice for 5 min. The mixture was then treated with 100 μg/ml PK on ice for 20 min. Digestion was stopped using 2 mM PMSF. The EMMs were harvested *via* centrifugation and washed with SEM buffer containing 2 mM PMSF. After washing with SEM buffer, EspP-inserted EMM proteins were collected by centrifugation, resuspended in SDS-PAGE sample buffer containing 2 mM PMSF, and analyzed by SDS-PAGE and radioimaging.

### Homotrimeric proteins (OmpC, OmpF, and LamB) assembly assay

For each point, EMM 150 μg pellets were resuspended in 200 μl assembly assay medium supplemented with 20 μl ^35^S-labeled OmpC, OmpF, or LamB. The assembly mixtures were incubated at 30 °C for 10, 30, 60, or 90 min. The assembly reactions were halted by heating on ice for 5 min. The EMMs were then harvested by centrifugation at 14,000 rpm for 5 min at 4 °C and resuspended in SEM buffer using a pipette. After SEM buffer wash, trimeric protein–assembled EMM pellets were solubilized in 1.5% DDM containing BN-PAGE lysis buffer on ice for 20 min. Solubilized proteins were clarified by centrifugation at 15,000 rpm for 10 min at 4 °C, followed by BN-PAGE analysis, as described above.

### CirA EMM folding assay

For each point, 70 μg of EMM pellets was resuspended in 100 μl assembly assay buffer supplemented with 10 μl ^35^S-labeled CirA. The assembly mixtures were incubated at 30 °C for 10, 30, or 90 min. At the indicated time points, the mixtures were placed on ice for 5 min. The EMMs were then harvested *via* centrifugation and resuspended in SEM buffer by pipetting twice. CirA-folded EMM proteins were collected *via* centrifugation. To prevent unintentional unfolding of CirA by heat, the pellets were resuspended in SDS-PAGE sample buffer at 16 °C and subjected to SDS-PAGE at 4 °C. Finally, the assembled protein levels were analyzed by SDS-PAGE and radioimaging. The peptide 23 competition assay was performed as described in the OmpT section.

### Densitometry and statistical analysis

For the EMM assembly assay and steady-state level protein comparison, three independent experimental datasets were densitometrically measured using the ImageQuant TL software from Cytiva. Statistical analyses were based on three independent technical replicates. Differences were analyzed by two-tailed Student's unpaired *t* test and considered statistically significant for *p* < 0.05. Exact *p* values of each experiment were described in the figure legends.

### *In situ* photocrosslinking

BL21 (DE3)∗ or Δ*bamE* were transformed with both pEVOL-BpF and pTnT-H6A2bamA (XBpa). The transformants were cultured overnight in LB medium at 37 °C. Overnight cultures were then shifted to XB (1% NaCl, 1% tryptone, 0.1% yeast extract, 1 mM BPA) at 37 °C in dark conditions until *A*_*600*_ reached 1.00. The cultures were either half exposed to UV irradiation for 7 min or kept in the dark. Cells were harvested and then solubilized with 1% SDS buffer (50 mM Tris-HCl pH 8.0, 150 mM NaCl, 1% SDS). Total protein lysate was diluted with 0.5% Triton X-100 buffer (50 mM Tris-HCl, pH 8.0, 150 mM NaCl, 0.5% Triton X-100) and then incubated with Ni-NTA agarose. The resin was washed by 0.5% Triton X-100 buffer (50 mM Tris-HCl, pH 8.0, 150 mM NaCl, 0.5% Triton X-100), and then proteins were eluted using elution buffer (50 mM Tris-HCl, pH 8.0, 150 mM NaCl, 0.5% Triton X-100, 400 mM imidazole-HCl, pH 8.0). The eluted fraction was analyzed by SDS-PAGE and immunoblotting with anti-BamD or anti-BamA antiserum.

## Data availability

All data are contained within the manuscript.

## Supporting information

This article contains supporting information (30, 35, 60).

## Conflict of interest

The authors declare that they have no conflicts of interest with the contents of this article.
