# Supplementary material for: Categorization of Escherichia coli outer membrane proteins by dependence on accessory proteins of the β-barrel assembly machinery complex
Source: J Biol Chem. 2023 May 15;299(7):104821. doi: 10.1016/j.jbc.2023.104821 (PMC10300371; doi:10.1016/j.jbc.2023.104821)
Supplement: Supporting Figure S1 [file mmc1.pdf]

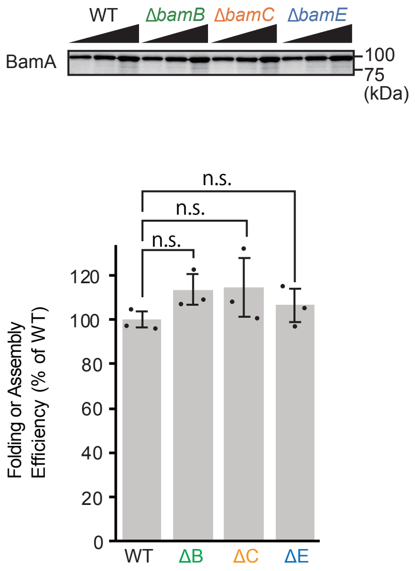

EMM fractions were isolated from WT and specified accessory protein deletion strains. BamA levels were quantified via SDS-PAGE and immunoblotting against BamA antibodies. EMM total protein levels were quantified as stated in text and loaded at 2, 4, and 8 μg of total EMM protein (black wedge). Densitometry analysis of 3 independent experiments displayed no significant difference between BamA levels across EMM samples. In all comparisons, the statistical advantage was not significant (n.s.), and the individual exact *P* values are shown follows, WT vs Δ*bamB* : *P* = 0.19475; WT vs Δ*bamC* : *P* = 0.31484; WT vs Δ*bamE* : *P* = 0.48227.

Thewasano et al.,2023 Supplemental Figure 1
