# Supplementary material for: Categorization of Escherichia coli outer membrane proteins by dependence on accessory proteins of the β-barrel assembly machinery complex
Source: J Biol Chem. 2023 May 15;299(7):104821. doi: 10.1016/j.jbc.2023.104821 (PMC10300371; doi:10.1016/j.jbc.2023.104821)
Supplement: Supporting Tables S2–S7 [file mmc2.docx]

**Tables**

**Table 2: Strains used in this study.**

| **E. coli strain name** | **Genotype** | **Reference**  **Or source** |
| --- | --- | --- |
| BL21 (DE3)* | F－, ompT, hsdSB(rB－ mB－), gal(λcI 857, ind1, Sam7, nin5, lacUV5-T7gene1), dcm(DE3) | Invitrogen |
| *ΔbamB* | BL21 (DE3)* Δ*bamB*::*kan* | Lithgow Lab |
| *ΔbamC* | BL21 (DE3)* Δ*bamC*::*kan* | Lithgow Lab |
| *ΔbamE* | BL21 (DE3)* Δ*bamE*::*kan* | Lithgow Lab |

**Table 3: Plasmids for *in vitro* transcription / translation.**

| **Plasmid name** | **Synthesized protein** | **Vector** | **Primers for construct** | **RE Site** | **Template DNA, source, or method** |
| --- | --- | --- | --- | --- | --- |
| pTnT-OmpA | OmpA | pTnT | -- | XhoI/XbaI | (35) |
| pTnT-OmpT | OmpT | pTnT | pTnTOmpT-f / pTnTOmpT-r | XhoI/XbaI | K-12 gene, SLiCE |
| pTnT-EspP | EspP | pTnT | -- | XhoI/XbaI | (35) |
| pTnT-OmpC | OmpC | pTnT | -- | XhoI/XbaI | (30) |
| pTNT-OmpF | OmpF | pTnT | -- | XhoI/XbaI | (35) |
| pTnT-LamB | LamB | pTnT | -- | XhoI/XbaI | (35) |
| pTnT-CirA | CirA | pTnT | pTnTLamB-f / pTnTLamB-r | XhoI/XbaI | K-12 gene, SLiCE (60) |

**Table 4: Plasmids for *in situ* photocrosslinking**

| **Plasmid name** | **Expressed protein** | **Vector/Promoter** | **Template DNA, source, or method** |
| --- | --- | --- | --- |
| pTnT BamA44 amb | BamA(L44BPA) amb-His6A2 | pTnT/BamA | (30) |
| pTnT BamA111 amb | BamA(K111BPA) amb-His6A2 | pTnT/BamA | (30) |
| pTnT BamA351 amb | BamA(K351BPA) amb-His6A2 | pTnT/BamA | pTnT-H6A2bamA, Quick change |
| pTnT BamA372 amb | BamA(M372BPA) amb-His6A2 | pTnT/BamA | pTnT-H6A2bamA, Quick change |

**Table 5: Primers for construction of deletion strains**

| **Primer name** | **Sequence 5'->3'** |
| --- | --- |
| BamB-f | gttgccgacctgcgtgg |
| BamB-r | gattttcctacgttagggcgcc |
| BamC-f | cacaacaaactatttgtcgaaccc |
| BamC-r | cgccttatccgaactacgtcc |
| BamE-f | cctgcttcacggtcagagtaaac |
| BamE-r | catttacagccgtccggc |

**Table 6: Primers for construction for *in vitro* transcription / translation**

| **Primer name** | **Sequence 5'->3'** |
| --- | --- |
| pTnTOmpT-f | pTnTOmpT-r ccgcccgggtcgactctagaTTAAAATGTGTACTTAAGACCAGCAGTAGT |
| pTntOmpT-r | pTnTOmpT-SLf tgttctttttgcactcgagATGTCTACCGAGACTTTATCGTTTACTCCTGACAACATAAA |
| pTnTCirA-SLf | tgttctttttgcactcgagATGGTCGATGATGATGGCGAAACGATGGTTGTCACTGCATC |
| pTnTCirA-SLr | ccgcccgggtcgactctagatcaGAAGCGATAATCCACTGCCATAAAGTAACGACGTC |
| pTnT-f | ACTTAATACGACTCACTATAGGCTA |
| pTnT-r | GGATCCAAAAAACCCCTCAAGACCC |

**Table7: Primers for *in situ* photocrosslinking**

| **Primer name** | **Sequence 5'->3'** |
| --- | --- |
| BamAK351amb-f | ccgtttctacgtgcgttagatccgttttgaaggta |
| BamAK351amb-r | taccttcaaaacggatctaacgcacgtagaaacgg |
| BamAM372amb-f | tcgcgaaatgcgtcagtaggaaggtgcatggctgg |
| BamAM372amb-r | tcgcgaaatgcgtcagtaggaaggtgcatggctgg |
